# Increase in the risk of clopidogrel resistance and consequent TIMI flow impairment by DNA hypomethylation of CYP2C19 gene in STEMI patients undergoing primary percutaneous coronary intervention (PPCI)

**DOI:** 10.1002/prp2.738

**Published:** 2021-02-28

**Authors:** Renan Sukmawan, Erick Hoetama, Siska Suridanda Danny, Astuti Giantini, Erlin Listiyaningsih, Vidya Gilang Rejeki, Amir Aziz Alkatiri, Isman Firdaus

**Affiliations:** ^1^ Cardiology and Vascular Medicine Department Faculty of Medicine Universitas Indonesia National Cardiovascular Center Harapan Kita Jakarta Indonesia; ^2^ Clinical Pathology Department Faculty of Medicine Universitas Indonesia Dr. Cipto Mangunkusumo National Public Hospital Jakarta Indonesia; ^3^ Indonesian Cardiovascular Research Center National Cardiovascular Center Harapan Kita Jakarta Indonesia

**Keywords:** acute coronary syndrome, clopidogrel resistance, CYP2C19, DNA methylation

## Abstract

Clopidogrel resistance is an important risk factor of ischemic event recurrence after optimal antiplatelet therapy. This study aims to investigate the role of CYP2C19 gene DNA methylation as one of the epigenetic factors for the risk of clopidogrel resistance in STEMI patients undergoing PPCI. ST‐segment elevation myocardial infarction (STEMI) patients undergoing PPCI were pretreated with clopidogrel, and their platelet function was measured using VerifyNow™ assay. The criteria for high on‐treatment platelet reactivity (HPR) were defined according to the expert consensus criteria (PRU >208). DNA methylation of the CYP2C19 gene was performed using bisulfite genomic sequencing technology. Furthermore, clinical, laboratory, and angiographic data including TIMI flow were collected. Among 122 patients, clopidogrel resistance was found in 22%. DNA methylation level percentage was lower in the clopidogrel resistance group (76.7 vs. 88.8, *p*‐value .038). But, the <50% methylation group was associated with increased risk of clopidogrel resistance (OR =4.5, 95%CI =2.1–9.3, *p*‐value = .018). This group was also found to have suboptimal post‐PCI TIMI flow (OR =3.4 95%CI =1.3–8.7, *p*‐value =.045). The lower DNA methylation level of the CYP2C19 gene increases the risk of clopidogrel resistance and subsequent poorer clinical outcome.

**Abbreviations**
ACS: acute coronary syndromes
HPR: high on‐treatment platelet reactivity
PPCI: primary percutaneous coronary intervention
STEMI: ST‐segment elevation myocardial infarction

## 1 INTRODUCTION

Dual antiplatelet therapy has become a standard regiment in the management of acute coronary syndromes (ACS).^1^ Despite the availability of new antiplatelet agents, clopidogrel is widely used in many circumstances. East Asian population has a significantly higher risk of bleeding with either prasugrel or ticagrelor compared with clopidogrel.^2^ In patients already treated with aspirin and clopidogrel, recurrent ischemic events still occur in up to 8%.^3^, ^4^ Many studies have demonstrated the role of HPR as an important risk factor for cardiovascular events during treatment with clopidogrel.^5^ This effect leads to the failure of platelet inhibition by clopidogrel, a clinical phenomenon known as clopidogrel resistance. Several factors including genetic and nongenetic, potentially contribute to the variability of platelet response.^6^ Some of the nongenetic factors are compliance, comorbidities, and drug–drug combinations. Based on the genetic polymorphism of CYP2C19 gene, notably CYP2C19*2 and CYP2C19*3, the loss‐of‐function allele leads to a poor metabolizer phenotype.^7^ These factors only partially explain the occurrence of clopidogrel resistance, and current studies have now shifted to the role of epigenetic factors. DNA methylation is an important epigenetic marker including microRNA and histone modification.^8^ Most studies regarding DNA methylation were conducted in the cancer field as well as autoimmune and neurodegenerative diseases. Compared with single nucleotide polymorphism, there is only limited evidence of an association between DNA methylation and clopidogrel resistance.^6^ Jia et al.^6^, ^9^, ^10^ demonstrated the correlation between clopidogrel resistance and several genes such as P2Y12 and PON1, where their methylation level was associated with the degree of clopidogrel response, depending on the function of each gene. These results underline the role of DNA methylation and clopidogrel resistance. However, the relationship between CYP2C19 gene DNA methylation and clopidogrel resistance has never been addressed. Since CYP2C19 is the principal enzyme responsible for the clopiogrel biotransformation, it was postulated that DNA methylation of the CYP2C19 gene affects platelet responsiveness to the drug. Hence, this study aims to investigate whether DNA methylation of the CYP2C19 gene contributes to the development of clopidogrel resistance and a worse clinical outcome in ACS patients. It is expected to give a better understanding of the vast mechanisms of clopidogrel resistance.

## 2 MATERIALS AND METHODS

### 2.1 Study population

From 2019 to 2020, 122 patients with ACS ST‐segment elevation myocardial infarction (STEMI) were recruited at National Cardiovascular Center Harapan Kita Hospital. The inclusion criteria were (1) onset of chest pain ≤12 h; (2) had primary coronary intervention (PCI); and (3) before PCI, received 600 mg clopidogrel and 300 mg aspirin as a loading dose. The exclusion criteria were (1) already received fibrinolysin (2) were given other P2Y12 ADP receptor inhibitors; (3) recent or chronic clopidogrel treatment; and (4) concomitant treatment with glycoprotein IIb/IIIa inhibitor.

Ethical approval was obtained from the Ethics Committee at National Cardiovascular Center Harapan Kita. All the patients or caregivers were provided written informed consent.

### 2.2 Blood sample collection and platelet function test

Venous blood samples were collected in EDTA and citrate tubes. Standard biochemical parameters such as ureum, creatinine, blood glucose, and lipid profile were obtained. Platelet function was measured using VerifyNow P2Y12 assay (Accumetrics Inc.). The results were reported in P2Y12 reaction units (PRU), and the values greater than 208 were considered as clopidogrel resistance.^11^

### 2.3 DNA methylation assay

Human genomic DNA was extracted from peripheral blood mononuclear cells using the QIAamp DNA Blood Mini Kit (Qiagen). The first step was bisulfite conversion of DNA which deaminates unmethylated cytosines to form uracil using EpiTech Bisulfite Kits. The second was polymerase chain reaction (PCR) primer design using Methyl Primer Express Software v1.0 and 7500 Fast Real‐Time PCR System (Table 1). The last was primer optimization and analysis of melting profiles. Furthermore, the melting profiles of PCR products from the sample DNA were compared to those from fully methylated and unmethylated reference samples.

**TABLE 1:** Primer design

Primers	Forward	Reverse
(CYP2C19)	5ʹ TTAGTGAGATTTCGTGGGC 3ʹ	5ʹ ATACGTACACCCTACGAAAACC 3ʹ

### 2.4 Angiographic analysis

Coronary angiogram was analyzed to evaluate pre‐ and post‐PCI Thrombolysis in Myocardial Infarction (TIMI) flow grade, pre‐PCI angiographic thrombus grade, and procedural related complication such as dissection.

### 2.5 Statistical analysis

Continuous data with a normal distribution were presented as means ± standard deviation, and compared using independent T‐Test or Mann–Whitney. Categorical data were presented as numbers and percentages and also compared by the *X* ^2^ or Fisher's exact test. Logistic regression was utilized to determine clopidogrel resistance predictors and reduced final TIMI flow. A two‐tailed *p*‐value less than .05 indicated statistical significance, whereas the analyses were carried out by IBM SPSS Statistics 22.0 (SPSS Inc).

## 3 RESULTS

### 3.1 Patients’ characteristics

From May 2019 to April 2020, a total of 122 patients that met the inclusion criteria were included in the final analysis and 27 of them (22%) were resistant to clopidogrel. The baseline characteristics between clopidogrel resistance and nonresistance are presented in Table 2. Moreover, fragment GRCh38.p13 (Genome Reference Consortium Human Build 38 patch release 13) was used, which includes three CpG island sequences all located in the gene body. Only one sequence was analyzed and it gave an optimal result, whereas the median percentage of DNA methylation was 87.73%. CYP2C19 genotyping was performed to evaluate the potential modulating effect of *2 and *3 allelic variants of the CYP2C19 gene. Wildtype phenotype was observed in 62% of the patients, whereas heterozygous phenotype was in 36%. The least common variant was homozygous, found in less than 1%. Laboratory and echocardiography parameters are presented in Table 3.

**TABLE 2:** Patient characteristics

Variables	*n* = 122
Sex	
Male, *n* (%)	112 (91.8)
Female, *n* (%)	10 (8.2)
Age, y	52.89 ± 10.13^a^
Age group, *n* (%)	
<65 y	104 (85.2)
≥65 y	18 (14.8)
BMI (kg/m^2^)	25.33 ± 3.81^a^
<25, *n* (%)	57 (46.7)
≥25, *n* (%)	65 (53.3)
Systolic blood pressure, mmHg	132.84 ± 26.9^a^
Diastolic blood pressure, mmHg	79.17 ± 18.26^a^
Risk factors, *n* (%)	
Hypertension	67 (54.9)
Diabetes mellitus	35 (28.7)
Smoking	87 (71.3)
Dyslipidemia	21 (17.2)
Family history	15 (12.3)
PPI usage, *n* (%)	10 (8.2)
STEMI onset, hours	7.5 (1–12)^b^
TIMI risk score	3 (1–8)^b^
Killip class, *n* (%)	
I	106 (86.9)
II	9 (7.4)
III	1 (0.8)
IV	6 (4.9)

^a^ Data presented as mean ± standard deviations.

^b^ Data presented as median (min−max).

**TABLE 3:** Laboratory and echocardiographic parameters

Variables	Median (min‐max)
Laboratory value	
Hemoglobin (g/dl)	14.27 (8.1–19.5)
Hematocrit (%)	41.12 (13–58)
Leukocyte (µl)	14168.13 (5310–89600)
Thrombocyte (106 μl)	276.59 (155–493)
Ureum (mg/dl)	32.02 (11.1–160)
Creatinine (U/L)	1.06 (0.1–4.46)
eGFR (ml/min/1/73 m)	91.99 (14–79.1)
Blood glucose (mg/dl)	180.51 (2–512)
hs‐troponin T (ng/L)	1637.53 (14–2152)
Total cholesterol (mg/dl)	170.74 (97–280)
LDL cholesterol (mg/dl)	123.37 (37–221)
HDL cholesterol (mg/dl)	37.15 (4–120)
Triglyceride (mg/dl)	137.42 (57–331)
Verify Now (PRU)	153.45 (8–301)
DNA methylation CYP2C19 gene (%)	87.73 (0–100)
CYP2C19 polymorphism, *n* (%)	
Wildtype	76 (62)
Heterozygous *2/*3	44 (36)
Heterozygous *carrier* *2	30 (24)
Heterozygous *carrier* *3	14 (11)
Heterozygous mutant *2 and *3	1 (0.1)
Homozygous mutant *2 and *3	1 (0.1)

### 3.2 Correlation between DNA methylation and clopidogrel resistance

According to Table 4, DNA methylation percentage was lower in the clopidogrel resistance group compared to nonresistance (88.8 ± 22.7 vs. 76.7 ± 32.9, *p* = .03). The level of DNA methylation was further divided into two groups, namely <50% and ≥50% methylation. The percentage of patients that belong to <50% methylation group was higher in the clopidogrel resistance group (22.2 vs. 8, *p* = .047) than in nonresistance. To overcome the effect of confounding factors, logistic regression analysis was performed, whereas genetic and nongenetic factors were included. Apart from the DNA methylation level, several variables were found related to the occurrence of clopidogrel resistance. Those were CYP2C19 genetic polymorphism, proton‐pump inhibitor usage, and comorbidities (diabetes mellitus and chronic kidney disease). A separate analysis found sex as the only independent predictor of DNA methylation level, where males had a higher percentage (Table S1).

**TABLE 4:** The comparison of characteristics between clopidogrel resistance and nonresistance groups

Variables	Verify Now ≤208 PRU (clopidogrel nonresistance) (*n* = 95)	Verify Now >208 PRU (clopidogrel resistance) (*n* = 27)	OR (CI 95%)	*p* value	Adjusted OR (95% CI)	*p* value
Clinical characteristics						
Age >65 y, *n* (%)	13 (13.7)	5 (18.5)	0.7 (0.3–1.7)	.532		
Male, *n* (%)	88 (92.6)	24 (88.9)	0.6 (0.1–2.6)	.532		
BMI ≥25 kg/m^2^, *n* (%)	53 (55.8)	12 (44.4)	0.6 (0.2–1.5)	.297		
Hypertension, *n* (%)	54 (56.8)	13 (48.1)	0.7 (0.3–1.6)	.423		
Diabetes mellitus, *n* (%)	19 (20)	16 (59.3)	5.8 (2.3–8.5)	<.001	6.2 (2.0–16.7)	<.01
Smoking, *n* (%)	69 (72.6)	18 (66.7)	0.7 (0.3–1.8)	.545		
Dyslipidemia, *n* (%)	16 (16.8)	5 (18.5)	1.1 (0.3–3.4)	.839		
Chronic kidney disease, *n* (%)	10 (10.5)	12 (44.4)	6.8 (2.4–12.5)	<.001	4.0 (1.2–12.8)	.019
Family history, *n* (%)	12 (12.6)	3 (11.1)	0.8 (0.2–3.3)	.832		
PPI usage, *n* (%)	4 (4.2)	6 (22.2)	6.5 (1.6–14.1)	.003	6.5 (1.3–15.9)	.025
Laboratory value						
Hematocrit (%)	42.1 ± 5.6	38.4 ± 5.9	—	.005		
Thrombocyte (106 µl)	274.7 ± 67.5	283.0 ± 62.6	—	.573		
Blood glucose (mg/dl)	176.5 ± 90.3	202.5 ± 80.8	—	.180		
CYP2C19 polymorphism, *n* (%)					3.4 (1.1–11.5)	.029
Wildtype	62 (65.3)	14 (51.9)				
Hetero/homozygous *2 and/or *3	33 (34.7)	13 (48.1)	1.7 (1.1–7.4)	.04		
DNA methylation CYP2C19 gene (%)						
Methylation<50%, *n* (%)	8 (8.4)	6 (22.2)	3.1 (1.9–6.9)	.037	4.5 (2.1–9.3)	.018
Methylation ≥50%, *n* (%)	87 (91.6)	21 (77.8)				

### 3.3 Factors affecting post‐PCI TIMI flow

Post‐PCI TIMI flow was divided into two groups, namely TIMI flow 3 and <3. Table 5 showed that the percentage of DNA methylation in the group with suboptimal post‐PCI TIMI flow was lower compared to the one with flow 3 (89 ± 22.6 vs. 79 ± 30.7, *p* = .054). Moreover, the percentage of patients that belong to <50% methylation group was higher in suboptimal post‐PCI TIMI flow than in flow 3 (21.6 vs. 7.1, *p* = .020). Clopidogrel resistance as defined by VerifyNow to be >208 PRU was also found as a predictor of suboptimal post‐PCI TIMI flow. Logistic regression analysis combining clinical and procedural factors are other predictors for suboptimal final TIMI flow which include the presence of diabetes mellitus, preprocedural TIMI flow ≤1, Kilip Class >I and STEMI onset >3 h.

**TABLE 5:** The Comparison of Characteristics between TIMI Flow 3 and TIMI Flow <3 Groups.

Variables	TIMI flow 3 (*n* = 85)	TIMI flow <3 (*n* = 37)	OR (CI95%)	*p* value	Adjusted OR (CI95%)	*p* value
Clinical characteristics						
Age >65 y, *n* (%)	14 (16.5)	4 (10.8)	0.6 (0.1–2)	.418		
Male, *n* (%)	77 (90.6)	35 (94.6)	1.8 (0.3–9.1)	.458		
BMI ≥25 kg/m^2^, *n* (%)	40 (47.1)	25 (67.6)	2.3 (1.0–5.2)	.037		
Hypertension, *n* (%)	47 (55.3)	20 (54.1)	0.9 (0.4–2.1)	.899		
Diabetes mellitus, *n* (%)	22 (25.9)	18 (48.6)	2.7 (1.2–6.1)	.014	7.8 (2.2–13.9)	<.001
Smoking, *n* (%)	61 (71.8)	26 (70.3)	0.9 (0.4–2.1)	.867		
Dyslipidemia, *n* (%)	13 (15.3)	8 (21.6)	1.5 (0.5–4.0)	.395		
Family history, *n* (%)	9 (10.6)	6 (16.2)	1.6 (0.5–4.9)	.384		
Killip class II–IV, *n* (%)	7 (8.2)	9 (24.3)	3.5 (1.2–10.5)	.016	2.1 (1.1–−6.3)	.048
STEMI onset, *n* (%)					1.8 (1.1–5.9)	.023
≤3 h	15 (17.6)	1 (2.7)				
>3 h	70 (82.4)	36 (97.3)	1.4 (1.2–1.7)	.025		
Anterior infarct, *n* (%)	54 (63.5)	21 (56.8)	1.3 (0.6–2.9)	.48		
EF <50%, *n* (%)	65 (76.5)	23 (62.2)	0.5 (0.2–1.1)	.105		
Laboratory value						
VerifyNow >208 PRU, *n* (%)	16 (18.8)	11 (29.7)	1.8 (1.1– 4.4)	.018	2.6 (1.6–7.5)	.038
Hematocrit (%)	40 ± 6.1	42 ± 5.1		.037		
Thrombocyte (106 µl)	281 ± 65.2	265 ± 68.2		.235		
Leukocyte (µl)	14211 ± 8744.3	13124 ± 4605.2		.519		
Blood glucose (mg/dl)	187 ± 96.8	170 ± 64.5		.32		
eGFR <60 ml/kg/1.63 m, *n* (%)	14 (16.5)	8 (21.6)	1.3 (0.5–3.6)	.496		
DNA methylation CYP2C19 gene (%)	89 ± 22.6	79 ± 30.7		.054		
Methylation <50%, *n* (%)	6 (7.1)	8 (21.6)	3.6 (1.1–11.3)	.020		
Methylation ≥50%, *n* (%)	79 (92.9)	29 (78.4)			3.4 (1.3–8.7)	.045
CYP2C19 polymorphism, *n* (%)						
Wildtype	55 (64.7)	21 (56.8)	1.3 (0.6–3.0)	.405		
Hetero/homozygous *2 and/or *3	30 (35.3)	16 (43.2)				
Procedural factors						
Infarct related artery, n (%)						
LM	4 (4.7)	1 (2.8)	0.5 (0.1–5.3)	.626		
LAD	77 (90.6)	31 (83.8)	0.5 (1.1–1.6)	.278		
LCx	31 (36.5)	11 (29.7)	0.7 (0.3–1.6)	.471		
RCA	50 (58.8)	19 (51.4)	0.7 (0.3–1.6)	.444		
Multivessel disease, n (%)	51 (60)	17 (45.9)	0.5 (0.2–1.2)	.151		
TIMI flow pre‐PCI, *n* (%)				.108		
0	55 (64.7)	27 (73)				
1	3 (3.5)	3 (8.1)				
2	8 (9.4)	5 (13.5)				
3	19 (22.4)	2 (5.4)				
TIMI flow pre‐PCI ≤1, *n* (%)	58 (68.2)	30 (81.1)	0.5 (0.1–1.2)	.146	4.9 (1.5–11.4)	.028
Thrombus *grade* >3, *n* (%)	85 (100)	35 (94.6)	3.4 (2.5–4.5)	.031		
Predilatation, *n* (%)	80 (94.1)	36 (97.3)	2.2 (0.2–19.9)	.455		
Thrombus aspiration, *n* (%)	3 (3.6)	2 (5.7)	1.6 (0.2–10.1)	.605		
Door to wire crossing	102 ± 77.5	93 ± 74.8		.526		
Procedural time (min)	47 ± 28.5	49 ± 27.2		.621		
Stent, *n* (%)	77 (90.6)	34 (91.9)	0.8 (0.2–3.3)	.817		
Dissection	1 (1.2)	1 (2.7)	2.3 (0.1–38.3)	.542		

## 4 DISCUSSION

### 4.1 Relationship between epigenetic, genetic, and nongenetic factors with clopidogrel resistance

Similar to previous studies by Tang et al.^12^ and Kathyrn et al.,^13^ it was confirmed that CGI location in the CYP2C19 gene lies within the gene body, meaning the gene's promoter region is poor in CpG (5ʹ‐Cytosine‐phosphate‐Guanine‐3ʹ). Kathyrn et al.^13^ stated that the CYP2C19 gene is possibly not the target of epigenetic control by DNA methylation. DNA methylation is known to exert an effect on a gene having abundant CpG island (CGI) on its promoter region. Other studies showed that DNA methylation tends to exhibit two different effects on gene expression.^8^ When this occurs at promoter regions, gene transcription process is inhibited. Conversely, DNA methylation in the gene body (intragenic DNA methylation) potentially manifests as active gene transcription and is known as the methylation paradox. Therefore, it also plays a role in CYP2C19 gene transcription regardless of the CpG poor status in its promoter region. The cut‐off point used for methylation status was 50% (hemi‐methylated), whereas 0% being unmethylated and 100% as methylated.^14^

CpG islands of CYP2C19 gene are located in gene body (intragenic) and according to the previous explanation about the methylation paradox, DNA methylation increases CYP2C19 gene transcription, but hypomethylation decreases it. Subsequently, less CYP2C19 gene expression occurs which decreases clopidogrel biotransformation and the active metabolites, therefore manifesting clopidogrel resistance.

The role of DNA methylation in repressing gene transcription in the promoter region has been extensively studied.^15^ There are three possible roles of intragenic DNA methylation, but the associated mechanism is not fully elucidated.^16^, ^17^ First, methylation silences repetitive DNA elements by blocking the initiation of their transcription, while also allowing normal transcription of the host gene to pass through them. Second, it prevents activation of transcription from internal promoters which are supposed to be inactive. Third, it regulates alternative splicing which is a process of selection and removal of multiple exon–intron in mature mRNA. More than 90% of human genes are subjected to an alternate splicing and its regulation is crucial for providing specific features for cells and tissues. From these three mechanisms, the importance of DNA methylation in the gene body besides the promoter region is appreciable. This potentially explains why CYP2C19 gene methylation level affects its expression and people's responsiveness to clopidogrel. However, there is the possibility that intragenic DNA methylation, such as in the CYP2C19 gene, is a consequence of other transcriptional regulation mechanisms rather than increased transcription level.^18^

Several studies by Jia et al.^6^, ^9^, ^10^ attempted to determine the relationship between DNA methylation of other genes and clopidogrel resistance. The genes were ATP‐binding cassette subfamily B member 1 (ABCB1), P2Y12, and paraoxonase 1 (PON1). As opposed to CYP2C19, CGI islands in those genes are located in the promoter region. DNA methylation in the promoter region inhibits the binding of transcription factor, therefore the gene expression is silenced.^19^ The P2Y12 study reported lower DNA methylation percentage in clopidogrel resistance group,^6^ however, that of PON1 found an opposite result.^9^ Lower methylation percentage of P2Y12 increases the gene's transcription, and consequently more P2Y12 receptors are available for ADP binding, which causes an increase in platelet activity. PON1 gene plays role in clopidogrel biotransformation. Hence, a higher methylation percentage decreases corresponding gene transcription, leading to less clopidogrel active metabolite. But, no significant relationship was found between ABCB1 gene DNA methylation and clopidogrel resistance.

Many factors influence DNA methylation status, such as demographics, diet, and environment.^20^ Sex was found as the only variable that has a significant difference in methylation level which is higher in males compared to females. The study by Osman et al.^21^ showed that males have higher levels of DNA methylation than females, even though the reason for this phenomenon is not fully explored. It is well recognized that DNA methylation is tissue‐specific, and using blood samples to determine its level might not give similar results.^22^ In terms of CYP2C19 (a liver enzyme), Kathryn et al. used the liver sample to measure DNA methylation level. However, Lindner et al. stated that DNA methylation in the liver being highly invasive is strongly reflected by DNA methylation in red blood cells. Therefore, it is possible to use a blood sample to measure CYP2C19 gene DNA methylation. Studies by Jia et al.^6^, ^9^, ^10^ also used such measurement methods. This supports the use of a blood sample as a fine substitute to demonstrate CYP2C19 gene DNA methylation level instead of using the human liver.

Aside from epigenetic factor, potential genetic, and nongenetic influence on clopidogrel resistance status were also investigated. CYP2C19 gene polymorphism (*2 and *3 loss‐of‐function alleles) was found in 48% patients with clopidogrel resistance and this phenotype increases the risk of the resistance by 4.2–5.3 times. The presence of *2 and *3 variants render the patients as intermediate or poor metabolizer. The same prevalence was found in other studies involving the East Asian population.^23^, ^24^ Furthermore, the positive relationship between CYP2C19 variants and platelet responsiveness to clopidogrel had been previously demonstrated.^25^ Since response to clopidogrel depends on its activation by CYP2C19, patients with either intermediate or poor metabolizer profile tend to exhibit less antiplatelet effect. The study by Mega et al.^26^ found that the aforementioned reduced‐function variants are responsible for lower plasma levels of clopidogrel active metabolites. This finding is essential considering that the prevalence of CYP2C19 polymorphism is higher in the Asian population.

Drug interaction with proton pump inhibitor (PPI) in pharmacodynamics and clinical study showed contradicting results,^27^ and this is owing to the inhibition of CYP2C19. Diabetes mellitus is a well‐known risk factor for inadequate response of antiplatelets.^28^ The mechanisms contributing to this phenomenon possibly has multiple factors, such as higher level of oxidative stress, procoagulants, and lack of sensitivity to insulin. All these factors cause impaired platelet P2Y12 receptor blockade responses. Another independent predictor found is the presence of chronic kidney disease (CKD). Hajimet et al.^29^ and Patrik et al.^30^ reported a higher prevalence of clopidogrel resistance in a population with CKD (defined as an estimated glomerular filtration rate of less than 60 ml/min/1.73 m^2^). The decreased effectivity of antiplatelet in CKD patients is partly due to less expression of CYP2C19, higher platelet turnover, increased procoagulants, and defect on the platelet as well as in prostaglandin metabolism.

### 4.2 Relationship between epigenetic, genetic, and nongenetic factors with post‐PCI TIMI flow

The causal relationship between DNA methylation and clinical outcome such as post‐PCI TIMI flow has not been addressed in previous studies. One possible mechanism is the increased prevalence of clopidogrel resistance. Capranazone et al.^31^ found that subjects with HPR seemed to have worse post‐PCI TIMI flow, higher thrombus grade, and longer corrected TIMI frame count. According to Aitmokhar et al.,^32^ no‐reflow phenomenon tends to occur in the HPR group. Both used the same assay and PRU cut‐off similarly to this study, including the population assessed. The presence of HPR has some consequence, such as high thrombus burden and increased production of intracoronary platelet‐derived microparticles (PMP). High thrombus burden increases the risk of distal embolization which then causes micro vessel obstruction, activation of coagulation and inflammation factors that lead to microvascular dysfunction, whereas PMP production triggers thrombus formation.

Diabetes mellitus is related to disturbance of coagulation/neurohormonal/autonomic function and endothelial dysfunction which increases vascular reactivity and thrombus formation.^33^ The longer the onset of STEMI, the larger the necrotic area that tends to occur. This increases the severity of microvascular edema and destruction, as well as the thrombus burden. Pre‐PCI TIMI flow of 2 or 3 indicates lesser thrombus burden, spontaneous lysis, better endogenous thrombolysis, and less vasospasm. Patients presented with more advanced Kilip class (II–IV), usually have lower ejection fraction which decreases coronary perfusion, more severe hemodynamic profile, and larger infarct size.

As best known, this study is the first to evaluate the relationship between CYP2C19 gene DNA methylation and clopidogrel resistance. It added further data to elucidate the role of intragenic DNA methylation since CYP2C19 gene DNA methylation is intragenic, and also evaluated the clinical outcome in terms of angiographic results. Compared to previous publications regarding DNA methylation and clopidogrel resistance, this study has the largest sample size. There were some limitations, first, from the three identified CGI fragments, only one can be analyzed. Second, additional possible confounding factors such as aspirin resistance and influence of other genes were not accounted.

## 5 CONCLUSION

In summary, it was indicated that DNA methylation of CYP2C19 gene plays an important role in the development of clopidogrel resistance apart from genetic influence such as polymorphism. The importance of epigenetic factors in the field of cardiovascular disease was also pointed out. Further studies with larger and more diverse populations in the genetic and epigenetic field are recommended for a better understanding.

## DISCLOSURE

The authors declare no conflict of interests regarding the publication of this paper.

## Supporting information

Table S1

## Data Availability

The data that support the findings of this study are available from the corresponding author upon reasonable request.
