# Supplementary material for: Increase in the risk of clopidogrel resistance and consequent TIMI flow impairment by DNA hypomethylation of CYP2C19 gene in STEMI patients undergoing primary percutaneous coronary intervention (PPCI)
Source: Pharmacol Res Perspect. 2021 Feb 28;9(2):e00738. doi: 10.1002/prp2.738 (PMC7915409; doi:10.1002/prp2.738)
Supplement: Supplementary file 1 — Table S1 [file PRP2-9-e00738-s001.docx]

| **Variables** | **CYP2C19 DNA methylation ≥50%** | **CYP2C19 DNA methylation <50%** | **OR (CI95%)** | **P value** |
| --- | --- | --- | --- | --- |
|  | **(n=108)** | **(n=14)** |  |  |
| Age >65 yrs, n(%) | 16 (14.8) | 2 (14.3) | 0.9 (0.1-4.7) | 0.958 |
| Male, n(%) | 102 (94.4) | 10 (71.4) | 0.1 (0.1-0.6) | 0.016 |
| BMI ≥25 kg/m2, n (%) | 58 (53.7) | 7 (50) | 0.8 (0.2-2.6) | 0.507 |
| Hypertension, n(%) | 62 (57.4) | 5 (35.7) | 0.4 (0.1-1.3) | 0.158 |
| Diabetes mellitus, n(%) | 31 (28.7) | 4 (28.6) | 0.9 (0.2-3.4) | 0.992 |
| Smoking, n(%) | 79 (73.1) | 8 (57.1) | 0.4 (0.1-1.5) | 0.223 |
| Dyslipidemia, n(%) | 20 (18.5) | 1 (7.1) | 0.3 (0.1-2.7) | 0.46 |
| Family history, n(%) | 14 (13) | 1 (7.1) | 0.5 (0.1-4.2) | 0.533 |
| Polimorphism of CYP2C19, n(%) |  |  |  |  |
| Wildtype | 67 (62) | 9 (64.3) | 0.9 (0.2-2.8) | 0.87 |
| Hetero/homozigous *2 and/or *3 | 41 (38) | 5 (35.7) |  |  |

**Supplementary Tabel**

**Table 1. The Comparison of Characteristics Between CYP2C19 Gene Hypomethylated and Hypermethylated Groups**
